# Genomic mutations and histopathologic biomarkers in Y^90^ radioembolization for chemorefractory colorectal liver metastases

**DOI:** 10.18632/oncotarget.25992

**Published:** 2018-08-21

**Authors:** Meaghan S. Dendy, Johannes M. Ludwig, Nima Kokabi, Stacey M. Stein, Jill Lacy, Howard S. Hochster, Hyun S. Kim

**Affiliations:** ^1^ Division of Interventional Radiology, Department of Radiology and Biomedical Imaging, Yale School of Medicine, New Haven, CT, USA; ^2^ Department of Diagnostic and Interventional Radiology and Neuroradiology, University Hospital Essen, University of Duisburg-Essen, Essen, Germany; ^3^ Division of Interventional Radiology and Image Guided Medicine, Department of Radiology and Imaging Sciences, Emory University School of Medicine, Atlanta, GA, USA; ^4^ Division of Medical Oncology, Department of Medicine, Yale School of Medicine, New Haven, CT, USA; ^5^ Yale Cancer Center, Yale School of Medicine, New Haven, CT, USA; ^6^ Rutgers Cancer Institute of New Jersey, New Brunswick, NJ, USA

**Keywords:** radioembolization, CRC, biomarker, colorectal liver metastases, CRLM

## Abstract

**Background:**

To investigate mutational load and histologic biomarkers as prognostic factors in patients with chemorefractory colorectal liver metastases (CRLM) treated with Y-90 radioembolization therapy.

**Materials and Methods:**

Single institution retrospective study of patients with CRLM who received Y-90 radioembolization after undergoing molecular testing was performed. Patient demographics, systemic therapy regimens, tumor characteristics and overall survival were analyzed between patients with differing histopathologic and genomic status. PIK3CA, KRAS, NRAS, AKT1, MEK1, MLH1, MSH2, MSH6 and PMS2 were analyzed. Kaplan-Meier survival estimation and multivariate Cox regression were analyzed.

**Results:**

23 patients underwent genomic analysis prior to Y-90. Eleven (47.8%) had mutations identified (MUT), and 12 were sequenced as wild type (WT) (52.2%). Median OS of 23 patients after Y-90 was 9.6 months (95% CI 6.67-16.23). Median OS from first Y-90 was significantly greater in WT patients (16.2 mo vs 6.5 mo; p =.0054). The survival difference between poorly differentiated tumors compared to all other histologic grades was significant (poor vs. well p=0.025, HR=26.8; poor vs. moderate p=.014, HR=23.07; poor vs. moderate/poor p=0.014, HR=23.68). When separated into 3 different groups (WT vs. MUT/moderate differentiation vs. MUT/poor differentiation) there was a difference in median OS observed (16.2 vs. 8.0 vs. 3.8 mos; p<.0001). Imaging response via RECIST criteria was significantly different between MUT and WT groups (p=0.02).

**Conclusions:**

Mutational status and histopathologic grade may predict survival after Y-90 radioembolization therapy for CRLM.

## INTRODUCTION

Patient management of colorectal liver metastases (CRLM) has improved over the past decade due to advances in surgical, medical and interventional radiologic treatments. Despite these advances, the prognosis of patients with metastatic colorectal cancer (mCRC) is estimated at 11.7% survival in the first 5-years, post-diagnosis [1]. Continued efforts are being made to establish genetic biomarkers that could predict treatment susceptibilities [2].

Genotyping CRC lesions has become standard of care in assessing prognosis of these patients and those lesions more susceptible to specific systemic treatments. Determining mismatch repair (MMR) protein mutational status has provided such predictive information in treating CRC patients. Lesions that are deficient in MMR proteins have been shown to be associated with a good prognosis [3]. These lesions have also been shown to be less susceptible to 5-fluorouracil therapy in the adjuvant setting [4]. In addition to MMR status, the analysis of KRAS, NRAS, and BRAF mutation status has become standard of care in CRC patient biopsies and determines whether patients will receive treatment with EGFR inhibitors [5, 6]. Integrating these analyses by using next-generation sequencing (NGS) to further outline the mutational load of individual tumors is now more common [7]. In utilizing this data, it has been hypothesized that those tumors with higher microsatellite instability (MSI-H) would be more susceptible to anti-PD1 immunotherapies [3].

Historically, the use of external beam radiation in treatment of CRLM has not been heavily utilized due to the paucity of data supporting efficacy in addition to radiation-induced hepatitis at levels > 30 Gy [8]. There have been some studies that show its use in control of pain in palliative treatment [9-12]. Stereotactic radiation has more recently established the delivery of greater doses of radiation in treating unresectable CRLM [13, 14]. Patients with CRLM commonly present for Y-90 radioembolization treatment in the salvage stage, after having failed systemic chemotherapy regimens, liver resection or ablation attempts. Data from multiple studies have shown the utility in treating chemorefractory CRLM with Y-90 radioembolization in combination with systemic chemotherapy or hepatic arterial infusion. Results from these studies showed increased time to progression, progression-free survival time and better response rate in those patients receiving Y-90 treatments [15-19].

This study aimed to evaluate the mutational status and histologic grade of mCRC lesions of the liver to determine patient outcomes after Y-90 radioembolization by comparing overall survival (OS) and progression free survival in a patient cohort receiving Y-90 for treatment of chemorefractory metastatic CRC of the liver.

## RESULTS

### Mutational status, histopathologic grade and disease characteristics

11 patients (47.8%) were found to have mutations. KRAS mutations were seen in 39% of patients, with BRAF, PIK3CA and hPMS2 mutations being identified in 4% of patients (Table 1). Patient demographics, tumor characteristics and systemic therapy regimen data are outlined in Table 2 between MUT and WT groups. Patients in the WT and MUT cohorts were similar in age, sex, race, pre-treatment tumor volume, index tumor size, primary tumor site, and mean pre-Y90 CEA level (all *p* > 0.05). Median time from primary CRC diagnosis to Y-90 treatment and also the time from liver metastasis to Y-90 treatment were shown to be significantly greater in the WT cohort when compared to the MUT group (36.17 mos vs. 15.27 mos; p=0.037 and 35.65 mos vs. 15.23 mos; p=0.025, respectively.)

**Table 1:** Tumor Mutations

Mutation	Patients (% of total cohort w/ MUT)
BRAF	4%
KRAS	39%
PIK3CA	4%
hPMS2	4%

BRAF= v-Raf murine sarcoma viral oncogene homolog B; KRAS= Kirsten rat sarcoma viral oncogene; PIK3CA= phosphatidylinositol-4,5-bisphosphate 3-kinase catalytic subunit alpha; hPMS2= Postmeiotic Segregation Increased 2.

**Table 2:** Patient Demographics and Disease Characteristics

Overall Characteristics		Wild-type [SD]	Mutant [SD]	p-value
Number of patients		12	11	-
Mean Age (years)		58.78; [12.40]	60.86; [11.98]	0.69
Sex	Male	8 (67%)	4 (36%)	0.86
	Female	4 (33%)	7 (64%)	
Race	White	10 (83%)	11 (100%)	0.3
	Black	0 (0%)	0 (0%)	
	Other	2 (17%)	0 (0%)	
Mean Pre-treatment Tumor Volume (cc)		142.43 [138.59]	227.86 [170.04]	0.3
Biopsy Sample Taken of Primary Tumor		11 (91.7%)	9 (81.8%)	0.94
Mean Index Tumor Size (cm)		5.35 [2.91]	6.2 [2.27]	0.49
Primary Tumor Site	Right Colon	1 (10%)	9 (90%)	0.83
	Left Colon	3 (37.5%)	5 (62.5%)	
Mean Pre-^90^Y CEA Level (ng/mL)		576.09 [852.05]	668.79 [1600.16]	0.88
Prior Resection of Primary		6 (50%)	5 (55%)	0.42
Ablation or Liver Resection Prior to Y-90		2 (16.7%)	3 (9.1%)	0.92
Mean Number of Systemic Therapies	Pre-^90^Y	3.5 [2.24]	3.36 [1.43]	0.86
	Post-^90^Y	2 [1.76]	0.9 [0.94]	0.08
	Total	5.5 [2.24]	4.27 [2.15]	0.19
**Median Time from Primary to ^90^Y (months)**		**36.17 [28.25]**	**15.27 [8.89]**	**0.037**
**Median Time from Liver** **Metastasis to ^90^Y (months)**		**35.65 [27.26]**	**15.23 [8.90]**	**0.025**

Bold font indicates significant p-values (<0.05).

CEA= carcinoembryonic antigen; KRAS= Kirsten rat sarcoma viral oncogene homolog; ^90^Y= Yttrium-90.

Histopathologic grade was shown to be well-differentiated in 4.3% of the cohort, moderately differentiated in 52.2%, moderately/poorly differentiated in 26.1%, and poorly differentiated in 8.7% of the cohort. Of the 23 patients, 2 did not have histopathologic grading available in the biopsy report.

### CEA response

CEA levels were evaluated within the 30 days prior to Y-90 radioembolization in all patients. Mean CEA before treatment for both WT and MUT groups were 576.09 ng/mL and 668.79 ng/mL, respectively (*p*=0.88). The mean change between CEA prior to Y-90 treatment and the CEA value obtained between 30-90 days post treatment was not found to be significantly different between WT and MUT groups (p= 0.43). Within the entire cohort, 25% were seen to have decreased CEA values of at least 50% post-treatment. There was a CEA decrease of less than 50% in 19% and 56% had increased CEA values after treatment. The difference in CEA response between WT and MUT was not significant (p=0.69).

### Survival

**Figure 1:**
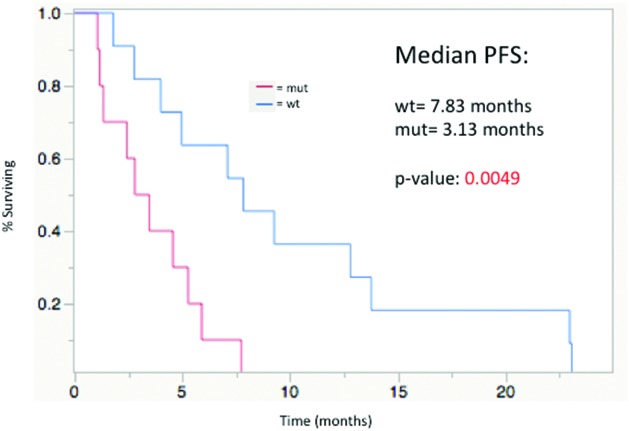
Progression-free survival from time of first Y-90 radioembolization treatment, stratified by mutational status; median PFS: WT= 7. 83 mos, MUT= 3.13 mos (p=0.0049)

Median progression-free survival (PFS) from the time of first Y-90 radioembolization was 4.96 months in the entire cohort. There was a significant difference in PFS between WT and MUT groups (7.83 mos vs. 3.13 mos; p=0.0049) (Figure 1).

**Figure 2:**
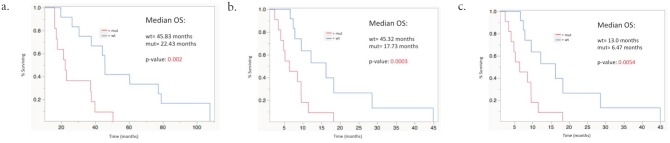
Median overall survival stratified by mutational status **(a)** From time of primary CRC diagnosis; p=0.002 **(b)** From time of liver metastasis diagnosis; p=0.0003 **(c)** From time of first Y-90 radioembolization; p=0.0054.

Median OS from the time of primary diagnosis, the time of liver metastasis diagnosis and the date of first Y-90 radioembolization were 37.8 months (95% confidence interval [CI], 22.43-45.8), 37.76 months (95% CI, 19.83-43.56) and 9.63 months (95% CI, 6.66-12.23). The Kaplan-Meier analysis showed a significant difference in median OS from the date of primary diagnosis, liver metastasis diagnosis and date of first Y-90 treatment in the WT group compared to the MUT group (p=0.002, p=0.0003, and p=0.0054, respectively) as seen in Figure 2a-2c. Median OS after Y-90 radioembolization was significantly prolonged in the WT group as compared to the MUT group (13.0 months vs. 6.46 months; p=0.0054).

**Figure 3:**
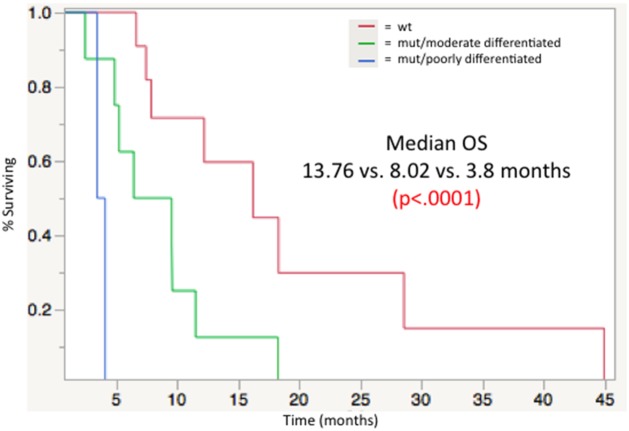
Median overall survival from time of first ^90^Y radioembolizaion, stratified by mutational load/histologic grade groupings: WT (13. 76 mos), MUT/moderately differentiated (8.02 mos), MUT/poorly differentiated (3.8 mos); p< 0.0001

There was a significant decrease in survival in patients with poorly differentiated tumors compared to all other histologic grades (poor vs. well p=0.025, HR=26.8; poor vs. moderate p=.014, HR=23.07; poor vs. moderate/poor p=0.014, HR=23.68). The cohort was then stratified into three groups: WT, MUT with moderate differentiated tumor histology, and MUT with poorly differentiated tumor histology. WT genotype group showed a significant survival advantage with a median OS of 16.2 months with MUT/moderate differentiation having a median OS of 8.02 months and MUT/poor differentiation being limited to a median OS of 3.8 months (p<.0001) (Figure 3).

**Figure 4:**
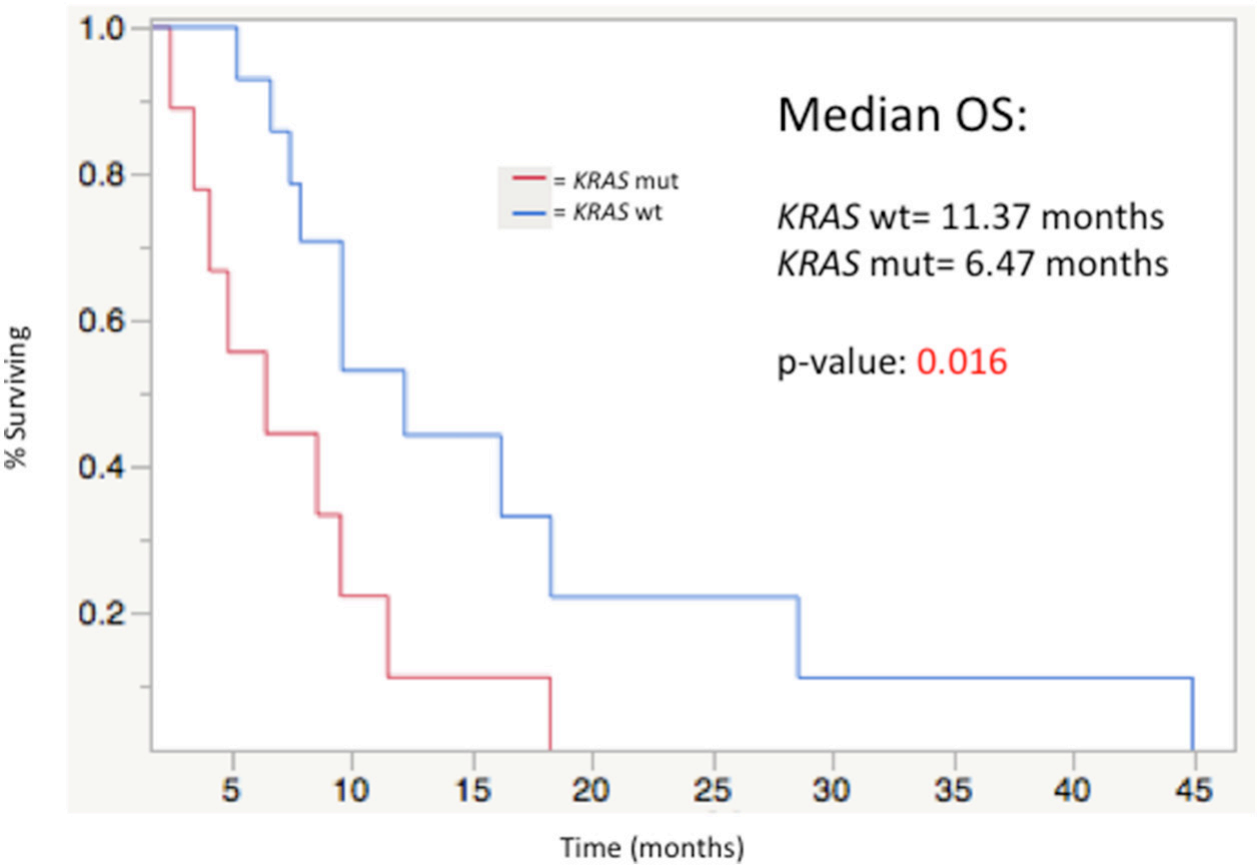
Median overall survival stratified by KRAS mutation status from time of first Y-90 radioembolization (MUT=11. 37 mos vs. WT= 6.47 mos; p= 0.016)

**Figure 5:**
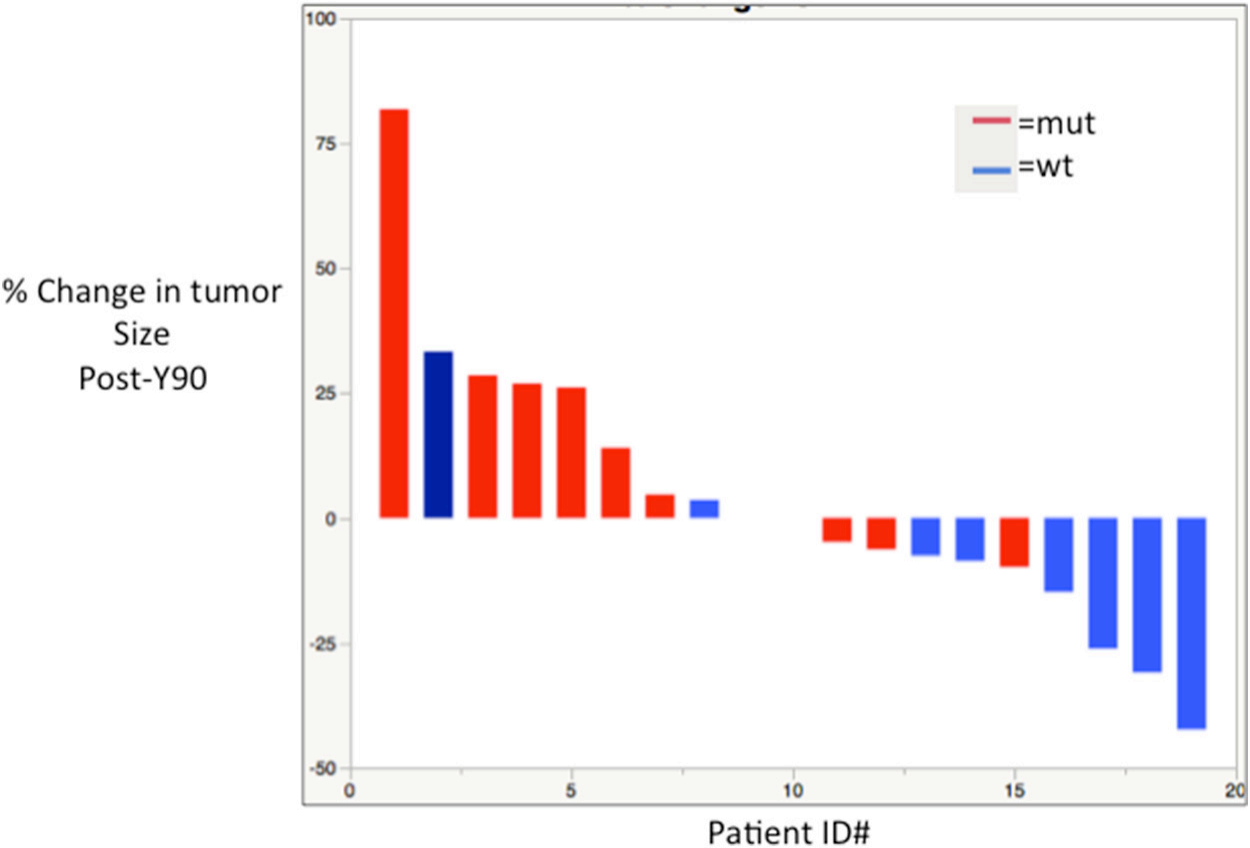
Waterfall plot showing response by % change in tumor size after Y-90 radioembolization

Univariate analysis of OS from the time of first Y-90 treatment demonstrated mutation in combination with histologic differentiation, mutations alone, KRAS mutation status (Figure 4) and RECIST response (Table 3, Figure 5) as prognostic factors for OS (Table 4). All other factors analyzed including age, sex, race, site of primary tumor, ECOG performance status, Child-Pugh Score, MELD score, EGFR Inhibitor Treatment (in the pre- or post-Y90 timeframe), pre-Y90 CEA, tumor size (cm), and tumor volume (cc) did not predict OS in WT compared to MUT groups.

**Table 3:** RECIST Response Stratified by MUT vs. WT grouping

	Response Parameter (RECIST)	% Response	ORR	p-value
Mutant	CR	0%	0%	**0.02**
	PR	0%		
	SD	44%		
	PD	56%		
Wild Type	CR	0%	27%	
	PR	27%		
	SD	45%		
	PD	27%		

Bold font indicates significant p-values (<0.05).

RECIST= response evaluation criteria in solid tumors; CR= complete response; PR= partial response; SD= stable disease; PD= progressive disease; ORR= overall response rate.

**Table 4:** Univariate Analysis

Prognostic Factor	Parameters	HR (95% CI)	p-value
Age	<65 yrs vs. >65 yrs	0.66 (0.188-1.86)	0.46
Gender	Male vs. Female	0.872 (0.336-2.165)	0.77
Race	White vs. Other	1.12 (0.175-4.045)	0.88
ECOG Status	ECOG=0 vs ECOG=1	1.67 (0.65-5.15)	0.30
Child-Pugh Score	Child-Pugh Score A vs. B	10.49 (0.49-109.5)	0.11
MELD	MELD ≤ 7 or > 7	0.92 (0.30-2.39)	0.87
Site of Primary	Right vs. Left Colon	2.25 (0.472-8.57)	0.28
Pre-^90^Y CEA	<100 ng/mL vs >100 ng/mL	2.133 (0.722-6.298)	0.166
Hepatic index tumor size	<5 cm vs >5 cm	1.85 (0.652-5.95)	0.25
Hepatic tumor volume	<100 cc vs >100 cc	1.61 (0.53-5.39)	0.402
EGFR Inhibitor Treatment	Systemic EGFR Inhibitor vs. None	0.42 (0.17-1.10)	0.07
Mut Status/Differentiation	WT vs. MUT/moderate vs. MUT/poor	5.068 (1.962-14.63)	**0.0008**
Mutation Status	WT vs MUT	0.255 (0.086-0.685)	**0.0066**
KRAS	WT vs MUT	0.314 (0.116-0.830)	**0.02**
Response (RECIST)	PD vs SD vs PR	2.44 (1.141-5.99)	**0.02**

Bold font indicates significant p-values (<0.05).

CEA= carcinoembryonic antigen; ECOG= Eastern Cooperative Oncology Group; MELD= Model for END-Stage Liver Disease; EGFR= Epidermal Growth Factor Receptor; RECIST= response evaluation criteria in solid tumors; KRAS= Kirsten rat sarcoma viral oncogene homolog; ^90^Y= Yttrium-90; PD= progressive disease; SD= stable disease; PR= partial response; HR= hazard ratio; CI= confidence interval.

### Multivariate cox regression

The prognostic factors for OS after Y-90 radioembolization found in univariate analysis were assimilated into multivariate Cox-regression modeling. Mutation status along with histologic differentiation was found to be an independent prognostic predictor of survival after Y-90 treatment (HR[95% CI]= 3.28 (1.097-10.45; p=0.0336)).

## DISCUSSION

The importance of optimizing patient selection for radioembolization treatment is paramount in yielding the best patient outcomes while simultaneously improving cost of patient care.

It has long been the standard of care to grade tumors utilizing criteria endorsed by the World Health Organization (WHO) and American Joint Committee on Cancer (AJCC). This utilization of tumor differentiation in treating patients has repeatedly been shown as a stage-independent prognostic marker [20]. In addressing the utilization of biomarkers to assess patient prognosis, the seventh edition of AJCC staging guidelines (AJCC-7) mentions new prognostic factors to be considered which include microsatellite instability, KRAS status and LOH 18q. These have not yet been added to current staging guidelines but may enhance future staging systems once incorporated [21]. Such observations are supported by the findings of this study in that jointly considering mutational data, along with histologic tumor assessment, can better predict patient outcomes after treatment of chemorefractory CRLM with Y-90 radioembolization. It should be noted that the prognostic differences observed due to the grade of differentiation is a well-established concept, as it has been repeatedly utilized in predicting local and distant disease growth. It has also been linked to mutant KRAS and a resulting undifferentiated phenotype in prior studies, which could explain the results of this study.

Recent studies have shown that various genetic and molecular biomarkers correlate with survival. Much research has focused on genetic biomarkers and heritable forms of CRC. Lynch syndrome results from inherited mismatch repair (MMR) gene mutations, most commonly consisting of MLH1, MSH2, MSH2, MSH6 and PMS2 [22]. Mutations in MMR genes lead to microsatellite instability and data have shown improved prognosis in these patients who commonly suffer fewer metastases after primary resection [23]. Further studies into the genetic makeup of CRC have analyzed the possible use in targeted therapies for patients with specific mutations. Two well-documented examples being KRAS and NRAS mutant tumors, which yield worse responses to anti-EGFR therapies [24, 25]. BRAF gene mutations have been shown to yield poorer prognoses, reducing both progression-free and overall survival when found in CRC biopsy specimens [26]. A recent study analyzed PIK3CA mutations and determined that there is no specific association between PIK3CA mutant CRC tumors and survival [27].

More recently, research has been completed on CRC biomarkers and how they may impact prognosis in those CRLM treated with Y-90 radioembolization. A recent review by our group outlined the research completed to date and found many imaging markers and some molecular markers that have been found and could inform decisions on which patients are appropriate for Y-90 treatment of CRLM [28]. There remains a lack of information in regards to genetic biomarkers that have been established as prognostic factors for Y-90 treatment of CRLM. KRAS was the initial genetic biomarker established in Y-90 treated lesions. The study showed that there was a significantly increased overall survival in those patients with WT KRAS tumor status when compared to those with mutant KRAS (9.5 mos vs. 4.8 mos; p=0.041) [29]. This finding was again shown by Magnetta et al., who showed that PFS was significantly prolonged in KRAS WT patients (166 days vs. 91 days; p=0.002) [30].

In this study cohort it could be demonstrated that grouping patients by mutation status, in combination with their histologic tumor grade, was shown to predict prolonged overall and progression-free survival (PFS) in those patients with WT status and well-differentiated tumors. It additionally supports prior studies that showed KRAS WT tumors receive increased survival benefit after receiving Y-90 radioembolization treatment. The response rate via RECIST was shown to be significant between MUT and WT cohorts in this study as none of the MUT patients yielded a complete or partial response after Y-90 treatment (WT=27% vs MUT=0% ORR; p=0.02).

There has been abundant research done on the phenomenon of radiation resistance in individual tumors. Unfortunately, very little has been focused on resistance to Y-90 radioembolization treatments specifically. One study by Janowski et al. looked at circulating cell-free DNA and showed that KRAS mutant CRLM yielded a smaller decrease in fragmentation index (FI) than the matched WT cohort after single lobe Y-90 treatment. This difference in FI was then shown to be associated with a significant increase in survival in the WT group (p=0.046) [31]. It is possible to further extrapolate from the current study potential explanations for resistance, in those patients with identified mutations, to Y-90 treatment. High levels of EGFR have been correlated with resistance to radiotherapy and also poor outcomes after clinical treatment [32, 33]. This is thought to be due to the pro-survival and pro-proliferation signals exerted by EGFR through the downstream PI3K/Akt, Ras/MAPK, and STAT pathways [34, 35]. The role of PI3K/Akt activity in radioresistance has been reported for various types of cancer, including lung, brain, and colon cancers. Mutant KRAS has been suggested to cause radioresistance in non-small cell lung cancer (NSCLC) by promotion of non-homologous end joining, though more research is needed to better establish this connection [36, 37]. A recent study published by Wang et al. demonstrates that the radioresistance of KRAS mutant NSCLC is likely due to EGFR-mediated chromatin condensation and that utilizing EGFR inhibitors in combination with ionizing radiation could yield better results in KRAS mutant patients [38]. Many more genetic associations have been made with regards to radiation resistance, though most of these mutations are not directly pertinent to those mutations described in this study. It should be noted that the “Mutated” cohort may have synchronous mutations in the genes analyzed making the resistance to radiotherapy unable to be explained solely by the mutational status of these lesions. Moving forward it would be of great interest to compare stereotactic radiation therapy in these patients to Y-90 in a prospective manner.

There are a number of limitations pertinent to this study. Firstly, this study was a retrospective data analysis. Secondly, the cohort size limits the power of the study’s statistical analysis. Another limitation is the type of sequencing utilized, as the retrospective nature of this study only allowed for some of the cohort to have the full-scale next-generation sequencing data available for analysis. Lastly, the genetically sequenced tumor biopsies collected from our patients were not all from the same site. This leaves the possibility of genetically heterogeneous tumors between primary and metastatic tumor sites. A large multi-institution prospective study would be greatly beneficial to validate mutational status and histologic grade as a prognostic factor in those with chemorefractory CRLM treated with Y-90 radioembolization.

## MATERIALS AND METHODS

### Patient characteristics and study design

This study was a retrospective review of 23 consecutive patients with genomic profiling treated with Y-90 radioembolization for unresectable chemorefractory CRLM as a third or fourth line therapy during the time period 2008-2016. These treatments were all administered at our tertiary cancer center and the study adhered to and was compliant with the Health Insurance Portability and Accountability Act (HIPAA) and had Institutional Review Board (IRB) approval.

Genomic mutation status was determined via review of surgical pathology reports from primary or metastatic tumor biopsy specimens collected prior to the first Y-90 treatment. Patients without genomic mutation data were excluded from further data analysis. Genomic mutation data was analyzed for PIK3CA, KRAS, NRAS, AKT1, MEK1, MLH1, MSH2, MSH6 and PMS2 genes. Histopathological grading was assessed by reviewing patient biopsy pathology reports. Patient demographics along with treatment course prior to Y-90 radioembolization were also assessed.

Overall, 23 patients (12 Males, 11 Females) who underwent genomic analysis prior to Y-90 radioembolization treatment for chemorefractory unresectable CRLM were included in this study. Median age of the cohort was 59 years (range=26-82 years.) All patients had been treated prior to Y-90 with systemic chemotherapy treatment and had shown progression of disease. Median number of chemotherapy treatment agents used prior to Y-90 was 3 (range=2-7 agents.) These chemotherapy regimens included: cetuximab, regorafenib, or panitumumab; oxaliplatin plus intravenous 5-fluorouracil (5-FU) and leucovorin with or without bevacizumab; irinotecan plus intravenous 5-FU and leucovorin with or without bevacizumab; oxaliplatin plus capecitabine. Patients were treated with resin-based Y-90 radioembolization with a mean administered activity of 27.29 mCi (range= 14.91-46.0 mCi.) Carcinoembryonic antigen (CEA) levels pre-treatment and up to 90 days after Y-90 radioembolization were documented and analyzed to assess treatment response. Pre-treatment and post-treatment imaging (3 months after first Y-90 treatment) were assessed for all 23 patients. Imaging consisted of CT or MRI, and pre- and post-treatment comparisons were only made using the same imaging modality as baseline assessment. Baseline and post-treatment tumor status were assessed along with the presence or absence of portal vein thrombosis. Treatment response evaluation was performed according to the RECIST 1.1 criteria [39]. Post treatment CEA Response was defined as an at least 50% decrease from pre-treatment CEA value within 30-90 days. Patient survival data and monitored progression were retrospectively collected up to March 2017.

### Treatment protocol

Radioembolization therapy was completed using a 3-F microcatheter and insoluble biocompatible resin Y-90 microspheres (SIR-Spheres, SIRTex Medical; Sydney, Australia). All patients first underwent shunt evaluation with technetium 99m macroaggregated albumin as in prior studies [40, 41].

Dosimetry was determined by assessing baseline tumor involvement via imaging. Computed tomography or magnetic resonance imaging volumes were analyzed using commercially available image processing software (MIM version 5.6.1; MIM software Inc.; Cleveland, OH, USA). Uniform liver tissue density was calculated as 1.03 g/cm^3^. The dosing was calculated using the body surface area method and adjusted for lung shunt fraction and also in those cases where the estimated lung dose was greater than 30 Gy per individual radioembolization treatment or cumulative dose of 50 Gy [42]. Lung shunt fraction was evaluated approximately 2 weeks prior to Y-90 therapy.

Radioembolization treatment was performed on an outpatient basis. All patients had bilobar disease and underwent consecutive treatments 1-month apart. Injection of SIR-Spheres was completed in an angiography suite using the standard technique previously described. Delivery of the calculated dose or vascular stasis were used as endpoints of microsphere injection [40, 41].

### Statistical analysis

Patient mutation status was correlated with demographics, laboratory markers, tumor characteristics, histopathologic stage, and chemotherapy regimens. These parameters were analyzed using Pearson χ^2^ and Student *t*-tests for categorical and continuous parametric data. Kaplan-Meier estimation and log-rank tests were used to compare overall survival (OS) from the date of primary CRC diagnosis, date of liver metastasis diagnosis, and the first Y-90 radioembolization treatment between patients with MUT and WT tumors. The same analysis was repeated for other prognostic factors tested.

Those prognostic factors found to be significant (*p* < 0.05) in univariate analysis were incorporated into a multivariate Cox regression analysis model. Statistical analyses were completed using JMP Statistical Software version 13.1 (SAS Institute Inc.; Cary, NC, USA).

## CONCLUSIONS

In conclusion, patients with chemorefractory CRLM treated with Y-90 radioembolization demonstrate significantly greater survival if they have a wild-type mutation status along with a well-differentiated tumor grade. Mutation status could be a useful prognostic tool in the decision to treat these patients with Y-90 radioembolization.
